# A Heuristic Angular Clustering Framework for Secured Statistical Data Aggregation in Sensor Networks

**DOI:** 10.3390/s20174937

**Published:** 2020-08-31

**Authors:** Lalitha Krishnasamy, Rajesh Kumar Dhanaraj, D. Ganesh Gopal, Thippa Reddy Gadekallu, Mohamed K. Aboudaif, Emad Abouel Nasr

**Affiliations:** 1 Department of IT, Kongu Engineering College, Tamil Nadu- 638060, India; klalitha.it@kongu.edu; 2 School of Computing Science & Engineering, Galgotias University, Uttar Pradesh 203201, India; d.rajeshkumar@galgotiasuniversity.edu.in (R.K.D.); dganeshgopal@gmail.com (D.G.G.); 3 School of Information Technology and Engineering, VIT-Vellore, Tamil Nadu 632014, India; thippareddy.g@vit.ac.in; 4 Advanced Manufacturing Institute, King Saud University, Riyadh 11421, Saudi Arabia; 5 Industrial Engineering Department, College of Engineering, King Saud University, Riyadh 11421, Saudi Arabia; eabdelghany@ksu.edu.sa; 6 Mechanical Engineering Department, Faculty of Engineering, Helwan University, Cairo 11732, Egypt

**Keywords:** clustering, radial-shaped clustering, node deployment, energy efficiency, routing, sensor networks

## Abstract

Clustering in wireless sensor networks plays a vital role in solving energy and scalability issues. Although multiple deployment structures and cluster shapes have been implemented, they sometimes fail to produce the expected outcomes owing to different geographical area shapes. This paper proposes a clustering algorithm with a complex deployment structure called radial-shaped clustering (RSC). The deployment structure is divided into multiple virtual concentric rings, and each ring is further divided into sectors called clusters. The node closest to the midpoint of each sector is selected as the cluster head. Each sector’s data are aggregated and forwarded to the sink node through angular inclination routing. We experimented and compared the proposed RSC performance against that of the existing fan-shaped clustering algorithm. Experimental results reveal that RSC outperforms the existing algorithm in scalability and network lifetime for large-scale sensor deployments.

## 1. Introduction

In most applications, sensors are used on a large scale to improve reliability and efficiency. A wireless sensor network (WSN) comprises a large number of sensor nodes with sensing and communication capabilities. The sensor nodes jointly collect and transmit data to the coordinator node, referred to as the sink node [1]. The main objective of deploying sensor nodes is to monitor the surrounding phenomenon, then process and transfer information to an analysis center. Sensor nodes are static, mobile devices commonly powered by limited power sources like batteries [2,3,4,5,6]. Sensor nodes have a limited transmission range. Consequently, optimal power utilization and the transmission of data over long distances is essential in a network of sensor nodes.

In a network of sensor nodes, clustering techniques are used for data collection, where sensor nodes are grouped into clusters for the conservation of energy [7]. Each cluster consists of nodes called members and a frequent reporting point called the cluster head (CH). Each node senses data and transmits them to their corresponding CH. Subsequently, the CH aggregates the collected data, converts them to a single tiny packet, and sends it to the sink node. Implementing the clustering technique reduces the network load and conserves energy, and makes the nodes live. 

Clustering, based on the size and shape of the deployment area, is a method adopted commonly to collect and transmit data to reduce energy consumption considerably. In previous research that has been studied, various clustering techniques have been identified to improve energy efficiency in a network of sensor nodes. However, most of the deployment structures adopted are either square or rectangular in shape. The square or rectangular-shaped structures might produce satisfactory results during simulation, but they are not suitable for all geographical regions for real-time deployment. 

In real-time applications, square-shaped sensors are not often deployed. For example, in airports, sensors are deployed in the passenger lobbies and runway paths, which are often irregular in shape. In weather stations also, sensors are deployed randomly in specific areas that are irregular in shape, based on the transmission range. Therefore, there is a need for an algorithm capable of adapting to any geographic region with different deployment structures. 

The security of sensor nodes and the data collected from the sensors that has to be transmitted securely to the sink or another network is another challenging issue, in addition to the deployment structure and cluster optimization. The intruders play a major role for spoofing the data in the middle. Many research algorithms and techniques were implemented to address the security issues like artificial intelligence mechanisms, multiagent schemes and artificial intelligent bots, etc. [8].

Although attention has been given to the deployment structure, energy optimization in sensor nodes poses a significant challenge in WSNs. In recent years, several clustering approaches have been aimed at addressing the efficient energy consumption issues in WSNs [4,5,9,10,11,12]. In most of the existing works, including LEACH [9], HEED [10], PEGASIS [13], enhanced LEACH and GBCR [14], and SPIN [15], the CH is selected based on the highest residual energy and the CH to CH data transmission is based on the coverage range and energy level. The selection and data transmission between CHs are based only on the energy levels and it could increase the number of hops from a CH to the sink node. The CH located closest to the mid area and angular-based routing can minimize the number of hops from the CH to the sink node. 

The major contributions of this work are:

- to determine the shape of the deployment area and the coverage range based on the network area to ensure that no node is left unattended and
- to select the CH with energy optimization to achieve a balanced WSN with minimal energy requirements.

## 2. Related Works

Clustering and routing are the fundamental processes in WSNs, and subsequent improvements have been made to the LEACH protocol. The cluster should cover all the nodes within the coverage area, and it should be provisioned with energy efficiency, marginal communication cost, and a balanced load in the network. Clustering protocols, such as LEACH, HEED, PEGASIS, TEEN, and APTEEN, focused on energy efficiency but failed to address the coverage of all the nodes deployed in the network [10,13,14,15,16]. 

The network area of a WSN is divided, based on the CH selected in LEACH, whereas it is an equal-sized grid structure in HEED. PEGASIS concentrates on transreceiving the data in local neighbor nodes instead of the CH, and the coverage of nodes was not discussed. Grid-based clustering was implemented in LPGCRA, GCMRA, and GCP [17,18] to facilitate network area coverage. In LPGCRA and GCP, the nodes are grouped based on their position; however, equal-sized grids could not be formed for randomly distributed nodes. 

Cluster chain-weighted metrics [19] achieve energy efficiency and increase network performance based on weighted metrics used to select a set of CHs. Ray and De [20] selected the CH using the k-means algorithm to prolong the overall network lifespan. The authors divided the process into three phases. The LEACH protocol is used to determine the initial CH selection. The network is partitioned into *k* clusters, and nodes join the nearest CH based on the Euclidean distance. However, the periodic reformation of clusters leads to network overhead. 

Agrawal and Pandey [21] proposed a CH election approach using fuzzy logic. Tentative CHs are elected in each round based on random numbers. The elected CH then uses two fuzzy parameters, which are at the local distance and of similar energy level. The local distance is the sum of all distances from neighboring nodes.

In a similar approach by Farman et al. [22], the network is partitioned into grids based on the node location where midpoints are computed using the membership degree. In the approach discussed by Meng et al. [23], two levels of square shaped grids are used to divide the network into high and low levels. For intracluster data transmission, a low-level grid is used, and for intercluster data transmission, a high-level grid is used. However, adopting this technique for large-scale deployment increases network complexity.

Moreover, topology construction is also vital for the distribution of nodes uniformly in the clusters or grids in case of grid-based approaches to make the network efficient. Although the deployment structure is considered as a grid, forming equal-sized grids in real-time deployment may not be feasible. Furthermore, the periodic reformation of clusters and the reselection of CHs could significantly increase energy consumption, thus leading to poor network performance [24,25,26].

To achieve reduced power utilization with minimal cost and congestion-aware routing, nodes are provisioned with transmission range adjustment, to reach the desired receiver. Transmission range adjustment and multihop forwarding are global positioning system (GPS)-based approaches. Considering the node’s radio and electrical energy, the combination of Geographic Adaptive Fidelity (GAF) and DGAF works well [27].

Lin et al. divided the network area into fan-shaped clusters (FSCs) and placed the sink node at the center of the fan-shaped coverage [28]. In the FSC, the diameter of the inner circle is twice that of the outer circle to balance the number of nodes in the cluster. The CH is selected randomly in each circle, and hierarchical energy level-based routing is followed. The sink node is located in the central area by default. As random CH selection in each circle increases the transmission distance, frequent CH selection is required for large-scale deployment. In real-time applications such as smart agriculture and natural environment protection, it may not be feasible for the sink node to be located in the central area. 

In this paper, the researchers propose an angular inclination-based clustering system to address the challenges of FSCs, which grabs attention in the high-volume deployment of sensor networks. This technique divides the high-volume network into virtual circles, and each virtual circle is further divided into various sectors called clusters to form radial-shaped groups. Moreover, angular inclination-based clustering has many benefits like minimizing the reclustering rate, ease of use, and robustness in routing, and accomplishes a balanced network load. 

## 3. Network and Energy Model

This paper adopted the radio energy model in HEED [10] for free space and multipath channel models. As the nodes are equipped with quasi-stationary distribution and scattered autonomously in a network field, the HEED’s energy model is considered to be the most appropriate choice. In addition to the built-in functionality of sensing nodes, the polar coordinates of each sensing node are known to that node, and sensing devices are GPS enabled. 

The sensing nodes are generally deployed independently and the distance is measured to connect the scattered nodes. After the outer structure is defined, the sensing field is divided into virtual circles. The diameter of the inner layer should be greater than that of the outer layers to avoid the hotspot issue. The network field is divided by using polar coordinates defined as follows:

- The radius of the circle (*r*)
- The angle (*θ*)
- The center position or midpoint of the area (*x*′,*y*′)

The sensing field is divided into circular-shaped clusters using polar coordinates of the circle, which uses the *r*, *θ*, and (*x*′,*y*′) based on the following formula:

(1) $$\theta =\frac{angle}{2}*i$$

(2) $$x=r*\cos\left(\theta \right)+x^{\prime }$$

(3) $$y=r*\sin\left(\theta \right)+y^{\prime }$$

**Figure 1:**
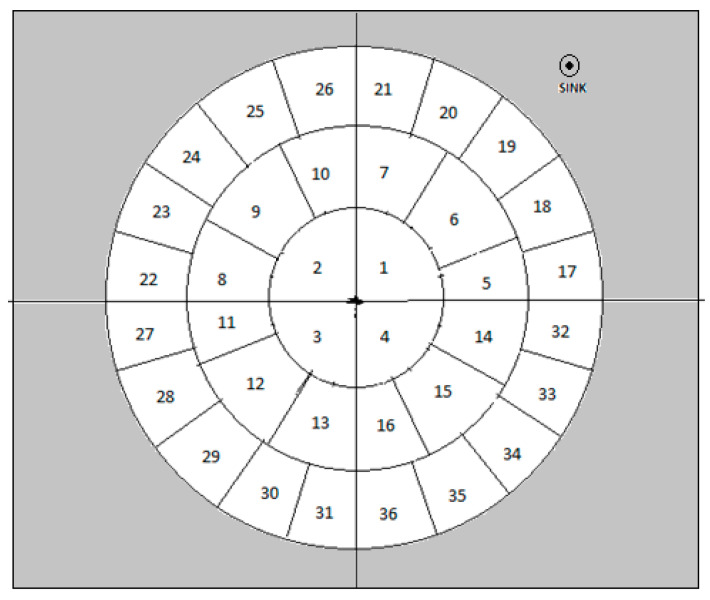
Deployment structure of radial-shaped clustering.

The deployment structure design is shown in Figure 1. The diameter of the first layer should be higher than those of the remaining outer layers. Each segment is further divided into equal-sized groups. Equal-sized groups contain randomly distributed nodes to achieve load balancing. The layers in the area are partitioned into (2∗i−1)∗n groups with n quadrants where i=(1,2,3,4…,m) is the layer from inner to outer structure and n=(2,4,6,8,…) represents the number of quadrants the area gets partitioned. 

Since the network field is portioned into concentric rings in fan shaped clustering by Lin et al. [28], the same layering and quadrants approach is adopted in this work. The quadrants are applied from 2 to 8 and dynamically changing the quadrant is beyond the scope of this paper. The Euclidean distance is used in HEED and applied to calculate energy requirements within the cluster region, and also the energy requirements for intercluster communication.

## 4. Radial-Shaped Geo Clustering and Angular Routing

The sensing nodes are deployed randomly over the deployment area, and the network area is divided into a circular-shaped virtual concentric m number of rings. Moreover, each virtual ring has k number of clusters. The sectors of radial-shaped virtual concentric rings *θ* are calculated from the midpoint of the network area $plot\;\left(x_{Center},y_{Center}\right)$ to the radius of each ring based on the sine and cosine transformations as given in algorithm lines 1 to 4. The nodes are deployed randomly and each node identifies its polar coordinates in line no. 8. The node identifies its own sector from lines 9 to 16 in Algorithm 1.
**Algorithm1.** Radial-shaped clustering (RSC).1:**Input:**r, m, $\left(x_{Center},y_{Center}\right)$2:**Output:** Total number of clusters3:k=(2∗ i−1)∗n 4:**for***i* = 1 to *k*5:  **for** *j* = 1 to *i*6:   Generate the number of sectors according to the Equations (1)–(3)7:   plot(x,y)8:   **end for**9:**end for**10:Deploy the nodes randomly and measure the polar coordinates by using the formula$r=sqrt\left(x^{2}+y^{2}\right)$$\theta =\mathrm{atan}\left(\frac{y}{x}\right)$11:**for***i* = 1 to s.no 12:***if***$node_{layer\left(i\right)}==r$13:   k=(2∗i−1)∗n14:       **for** *j* = 1 to *k*_nodes15:            **if** $node{\left(i\right)}_{angle}\leq sector{\left(i\right)}_{angle}$16:           node(i).position≔sector(i).position17:            **end if**18:       **end for**19:   **end if**20:**end for**

### 4.1. CH Selection in RSC

The clusters are partitioned in each sector of the radial-shaped virtual ring, and the nodes identify their position. For each cluster, a Euclidean distance is calculated for all the intracluster nodes as local coordinates, and the node closest to the midpoint of a cluster becomes the initial CH. Subsequently, the re-election process starts whenever the CH’s battery level is less than the set threshold. The sensing node with the highest battery level becomes the CH of the cluster. 

The procedure to select the CH is presented in Algorithm 2. The size of randomly deployed nodes Size (A), midpoints Size (Grp), side length of a cluster (a) and the radius (r) are taken as the input parameters. From the lines 1 to 4 in the Algorithm 2, the midpoint of the sector is taken as the minimum threshold value and calculates the *x*, *y* coordinates for each node to compare with the maximum of *x*, *y* coordinates in each sector. The lines Nos. 6 to 12 measure the radius, the angle and the distance of each node within the sector. The node which is nearest to the midpoint with maximum energy is taken as a CH in each sector according to the lines Nos. 15 to 19 in Algorithm 2. This procedure continues until all the CHs are selected in the deployment area.
**Algorithm2.** CH selection in radial-shaped clusters.1:**Input:** size(A), size(grp), a, r2:**Output:** Each cluster with CH3:**for***i* = 1 to Size(Grp)4:  Find the min. and max. of *x, y* coordinates for each sector with **a**5:  *k* = 1 6:  **for** *j* = 1 to Size(A) 7:   ***if*** min(x) ≤node.x≤max(x) and min(y) ≤node.y≤max(y)
8:     $C_{k}\left(i\right):=node.id$
9:     $radius_{C_{k}}=r/2$10:     $angle\left(C_{k}\left(i\right)\right)=\;\theta /2$11:     $Dist_{i}\left(j\right)=\;$ the distance between the node location12:     **end if**13:  **end for**14:**end for**15:**for***l* = 1 to node_count **do**16:     $Min_{i}\left(l\right)=\min\left(Dist_{i}\left(l\right)\right)$17:   ***if*** $\left(Min_{i}\left(l\right)==\;Min_{i}\left(l-1\right)\right)$18:          Verify the level of energy and select the node with max. energy      $Ch_{i}\left(k\right)=\;Min_{l}\left(node.id\right)$19:     **end if**20:   k = k + 121:**end for**

The CH sends data towards the next lower layer of the ring, which in turn sends the data to the next lower layer and so on. This action terminates when the sink node receives the data. The CH may also act as the relay node. In this strategy, the CH is assumed to be simple and efficient, which minimizes the intracluster communication and reclustering costs of the repetition process. 

Furthermore, the CH broadcasts its head message to all the other members of the clusters. First, it reduces the intracluster communication cost as the CH is located at the midpoint of the clusters. Second, the reclustering frequency is minimized as it happens only when no node is available in the midpoint of the cluster area.

### 4.2. Routing Model

In RSC, intracluster communication is established with single-hop routing. For intercluster communication, multihop or hierarchical routing is necessary, because the data from a cluster aggregated by the CH are transmitted to the sink node via other CHs in the structure deployed nearby. The sink node can be located anywhere within the network deployment, and the routing path is established based on the location of the sink node. Data transmission is a suboptimal process using traditional routing models based on the shortest path distance. We adopted chain-based routing to achieve optimal data transmission. Therefore, we propose a routing algorithm based on the angular inclination technique used in Lalitha et al. [17] mainly to transmit data toward the sink node. The primary objective is to identify an optimal routing path from every CH to the sink node, as implemented in [23,27,28,29]. 

To find the optimal path, a threshold angle is taken and the angle between the CH and the sink is calculated by finding the angular difference from lines Nos. 3 to 6 in Algorithm 3. Once the angle is measured, the distance between inter-CHs towards the sink is calculated from lines 7 to 11. Initially, neighbor CH is zero. After the distance and the angular measurement, the CH with minimum distance and maximum energy is selected as a next hop CH. This process is depicted in the algorithm from lines 17 to 23.
**Algorithm3.** Angular Routing in RSC.1:**Input:**α- Threshold value, CH_n_ –number of CHs2:**Output:** Optimal routing path to the Sink3:Find the angle amongst CH and the Sink4:**for**$\;Ch_{\left(i\right)}$=1 to CH_n_5:  $\theta =\tan^{-1}\left(\frac{y}{x}\right)$6:  β1= θ+ α  β2= θ− α7:  **for** $\;Ch_{\left(i\right)}$=1 to l8:   ***if*** $Ch_{\left(i\right)}\;!=\;Ch_{\left(l\right)}$9:      ***if*** $Ch_{\left(l\right)}.degree\geq \;\beta 2\;\&\&Ch_{\left(l\right)}.degree<\;\beta 1\;$10:            $path\left(\;Ch_{\left(i\right)}\right)=l$11:            $distance\left(ch_{\left(i\right)\;}\right)=Dist(ch_{\left(i\right)},ch_{\left(l\right)}$)12:          **end if**13:      **end if**14:  **end for**15:**end for**16:Neighbor($\;Ch_{\left(i\right)})$=017:**for** j=1 to $path\left(\;Ch_{\left(i\right)}\right)$18:  ***if*** $path\left(ch_{\left(i\right)},\;ch_{\left(j\right)}\right)!=0$19:     $\left(Min_{i}\left(j\right)==\;Min_{i}\left(j-1\right)\right)$20:            Find the node which has min. distance    $Min_{dst}\left(node.id\right)=\;Ch_{i}\left(j\right)$21:      **end if**22:**end for**23:$next_{hop\left(ch_{\left(i\right)}\right)}=Min_{dst}\left(node.id\right)$

In this angular routing, an angular structure is formed between two CHs, and the data are forwarded from one angular aligned CH to the other. It reduces the hop count, unlike the heuristic approach of finding the shortest path. Data forwarded from CH to CH are random in the LEACH protocol, and the chain model-based forwarding of data from one cluster member to another is implemented in PEGASIS. There is no assurance of reaching the sink node in the former technique and finding alternate routes is complex in the case of node failure in the latter. These problems are alleviated in the current routing technique that is applied in RSC.

## 5. Simulation and Performance Evaluation

The RSC algorithm was implemented using MATLAB 2013a and core i5 processor with Windows-7. The transmission radius of the sensor nodes was at 100 m. 

The experiment results were taken from twenty different scenarios like varying the number of nodes from 500 to 1000. The network field was assumed to be a virtual circular-shaped area where sensor nodes were distributed randomly. The number of nodes deployed and the number of rounds were changed frequently to obtain the cluster data for further analysis. The sink node was placed in the center of the circular area initially. The sink node location was changed randomly in different scenarios. The parameters used in MATLAB are defined in Table 1.

**Figure 2:**
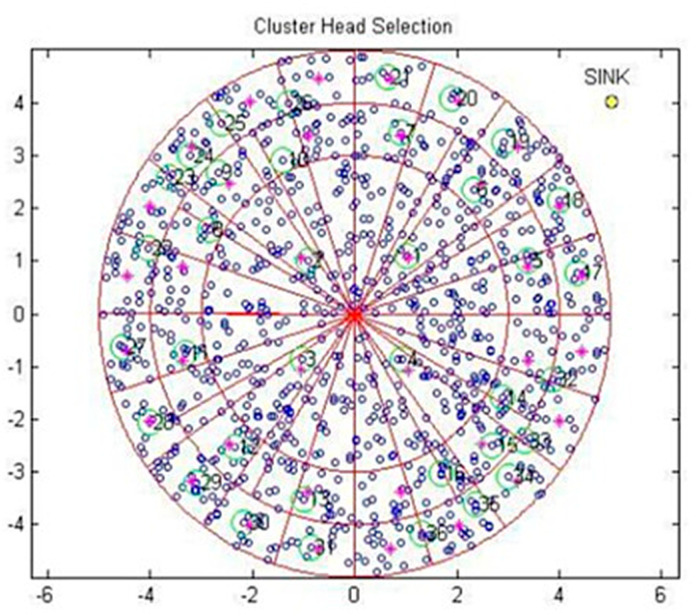
Node deployment and CH selection.

The cluster head selection in RSC is presented as an example scenario in Figure 2. The radius of the inner circle was twice that of the remaining circles to avoid the hotspot issue since all the communications were towards the sink location. The angle θ was taken as 90° for fixing the quadrant in this example scenario, hence n was 4 and each quadrant was taken as a single sector. Subsequently, the second layer was subdivided into three sectors by $\frac{\pi }{3}$. Furthermore, the next layer was subdivided into five sectors by $\frac{\pi }{5}\;$ for the third circle, and so on. The nodes were thrown randomly in the deployment area and each sector was considered as a cluster. Each cluster needed a CH to aggregate the data. 

The CH was identified by the minimum distance closest to the midpoint of the cluster, as shown in Lalitha et al. [17,18]. Initially, the sink was located at the center of the network field, and then it was changed dynamically to evaluate the performance. 

The process of routing takes place when the node forwards the data collected towards its CH. The probability of data transmitting to the nearest CH towards the sink node is a binomial polynomial problem. Angular inclination routing is one of the alternatives to ensure that the data can be reached to the Sink node. 

**Figure 3:**
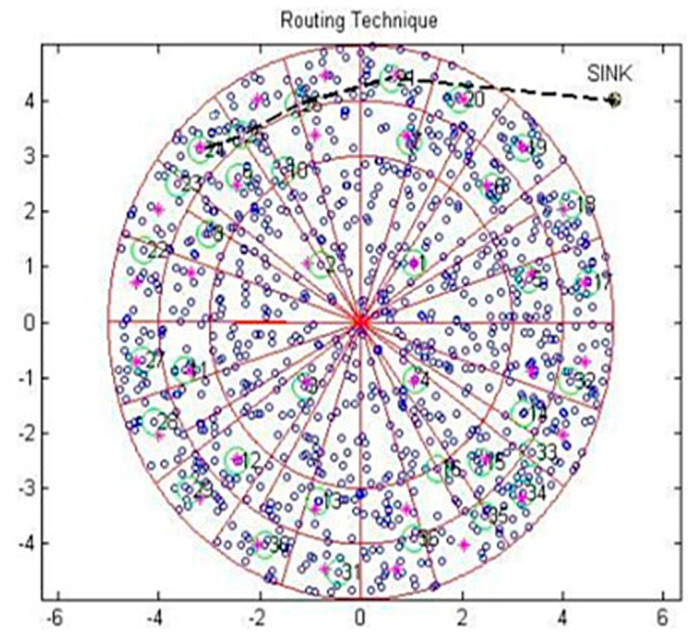
Routing for the RSC.

The CH transmitted the data towards the subsequent lower layer sensing node in the angular inclination routing, as discussed in [23,30]. This process continued toward the angle of the sink node until the data reached the sink node. The scenario presented in Figure 3 shows that the third layer of the CH wanted to transmit the data to the sink. The sink location was identified and a virtual path was framed from CH to the sink with 30-degree angular inclination. In this routing, the number of hops was greatly reduced and there was a moderate increase in the transmission range. 

### 5.1. Behavioral Analysis of the RSC

The performance of the RSC was analyzed based on the following criteria: the number of live nodes, the total network residual energy, and the packet received ratio.

1. **Live sensing nodes**: the total numbers of sensing nodes with an energy level greater than the threshold are called live sensing nodes.
2. **Total residualenergy**: this is the total energy level of all live sensing nodes measured in joules (J).
3. **Packetreceived ratio**: the packet received ratio for each round is calculated as the ratio of the total number of packets received and the node count.

The packet received ratio metric used statistical measures as referred to in Chen [31], are listed below:

- Mean
- Median
- Mode
- Maximum
- Variance or standard deviation

**Figure 4:**
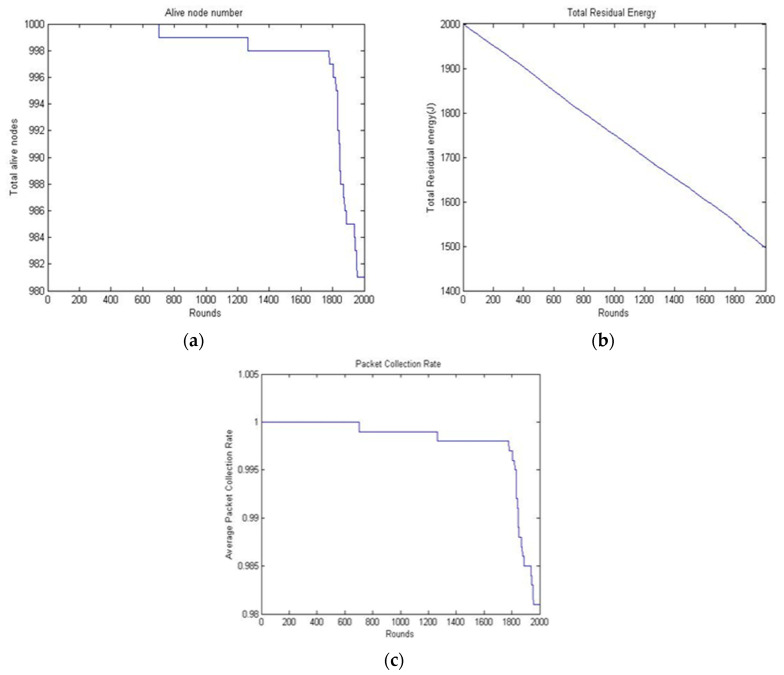
Behavioral analysis of RSC: (**a**) the number of live nodes; (**b**) the residual power level of RSC; and (**c**) the packet received ratio of RSC.

Figure 4 shows the number of live nodes, the residual power level of all the nodes, and the ratio of packets collected based on the time (number of rounds). As shown in Figure 4a, the number of live nodes decreased as the number of rounds increased. It was observed that all the nodes were alive until 700 s and started to die at about 1700 s. The energy level indicated that the proposed algorithm achieved improved load balancing. As shown in Figure 4b, the residual power level of the total deployed nodes decreased with the increase in duration. The sudden increase in energy consumption of a network was caused by the following: first, the nodes’ count expired after certain duration; the long route needed to be identified by the packets to reach the sink node. Secondly, the frequent re-election process. The energy level was expended rapidly in both cases. Figure 4c shows that the rate of packets received started to decrease at the duration of 700 s. It was observed that up to 700 s, the packet collection rate was 100% and then it decreased subsequently. Even after 2000 s, almost 50% of the nodes were still alive, which gave a packet collection rate of 50%. This indicated that the nodes were alive in the first layer and showed that the RSC achieved load balancing.

### 5.2. Comparison of RSC with Fan-Shaped Clustering

The RSC results were compared against the existing FSC in Lin et al. [28] to analyze the performance of RSC since both the algorithms applied angular clustering and aimed to increase energy efficiency and to reduce reclustering costs in large-scale networks. 

The existing algorithm FSC was compared with HEED to find the number of alive nodes with 2000 rounds, i.e., 2000 seconds and produced the results as 42% live nodes with more than 40% live nodes left in FSC compared to 1/3 of nodes with 10% energy in HEED. Since the existing work is already compared with the state-of-the-art algorithm, FSC alone was taken for comparison and simulation. Both RSC and FSC were executed up to 3500 rounds. 

Similarly, the existing work was compared with HEED for the evaluation of total residual energy and packet collection rate. In most of the scenarios, it was executed up to 2000 rounds. To achieve better results with long duration and large-scale network, the FSC was compared with RSC for 3500 rounds for the above two parameters.

**Figure 5:**
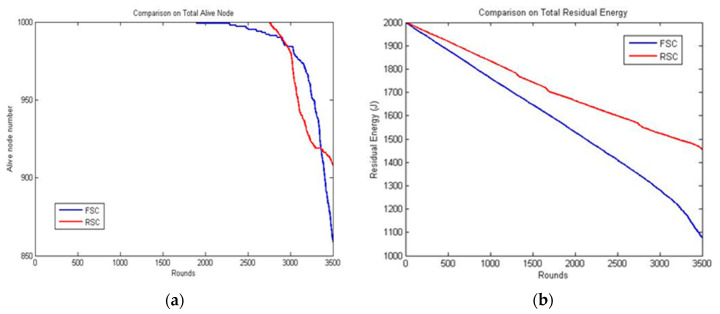
Comparison of RSC and FSC: (**a**) the number of live nodes and (**b**) the total residual energy.

The results of RSC and FSC were analyzed based on the total live nodes. Initially, the number of nodes was stable in both the cases. In FSC, the number of nodes started to decrease at 1800 rounds and reaches 860 at 3500 rounds. Conversely, in RSC, the number of nodes started to decrease at 2800 rounds and reached 920 at 3500 rounds. This indicated that the nodes started consuming energy evenly; they died at almost the same time. Figure 5a shows considerable load balancing up to 3000 rounds. The total residual energy decreased with time, as shown in Figure 5b, as the node had to find a route to reach the sink node and the reclustering process. RSC performed better because the transmission distance was less than that in FSC. 

The packet received ratio is considered as the most significant measure because it reveals packet loss and the dead nodes in the network. Packet loss may occur when

1. none of the nodes is selected as the CH,
2. the CH node could not find the forwarder to reach the sink node, and
3. the distance to reach the relay node is much greater.

The packet received ratio was analyzed using statistical measures considering the factors mentioned above and the large scale of the applications [29,32,33,34,35,36,37,38,39]. For example, the mean values were considered effective for real-time traffic applications, but event-based applications would prefer median and mode values. Some of the applications and preferred statistical measures were as follows:

- Mean—power distribution units
- Median—capacity planning or cost predictions
- Mode—power consumption of an entire network operation center
- Maximum—domestic applications/heat transfer
- Standard deviation and/or variance—to trigger a warning or an alarm if a system operates outside an acceptable range

**Figure 6:**
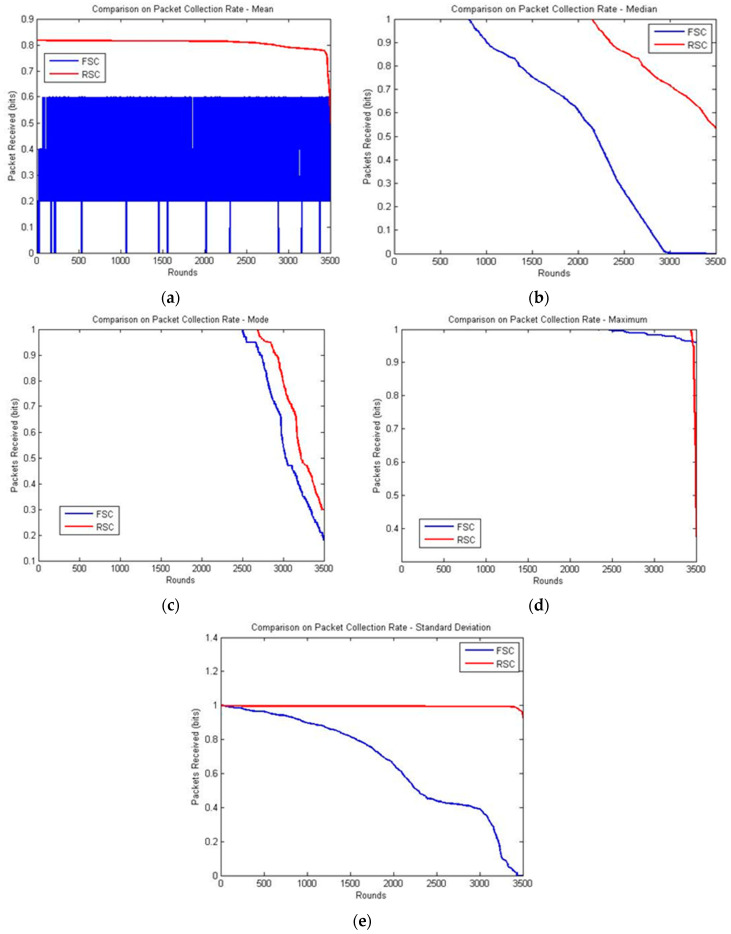
Comparison of the package collection rate: (**a**) mean; (**b**) median; (**c**) mode; (**d**) maximum; and (**e**) standard deviation.

The packet received ratio comparison of the FSC and RSC is shown in Figure 6. In FSC, the packets received were 0.6, whereas in RSC, the packets received were 0.82, and it started to decrease gradually to 0.8 at 2700 rounds and reached 0.5 at 3500 rounds based on the mean values. This is because the radius of the inner and outer layers is equal for RSC, but FSC produces an inner layer that is twice that of the outer layer, which increases the transmission distance. The uniform size of the layers in RSC constitutes a high mean and median packet collection rate, as shown in Figure 6a.

RSC and FSC produced results with slight deviations in the mode and maximum statistical measures. Both the algorithms aim to reduce the frequency of the reclustering cost, which was achieved at 2700 rounds in the FSC and 3000 rounds in RSC, as shown in Figure 6c,d. Subsequently, the packet collection rate decreased rapidly due to the finding of the relay node and transmission distance with the present live nodes.

The results of RSC and FSC in standard deviation showed that the size of a data set may vary according to the time and range. In a certain interval period between two grouping practices, the rate of packet collection reduces because of the nonexistence of CHs. If the variance measure is high, the deviation of data is also high, which leads to inadequate analysis. This is identified in FSC owing to the size of the central area. Data are transmitted from the outer layer to the inner layer until they reach the CH which is located in the central area. Frequent data collection leads to repeated reclustering, which decreases the packet collection rate. In the FSC, the initial packet received started decreasing gradually from the starting round and reached 0 at 3500 rounds, whereas in RSC, the initial packet received was 1.00, and it started to decrease gradually at 3400 rounds and reached 0. 85 at about 3500 rounds.

Figure 6e shows that the proposed system maintained its stability and achieved an improved packet collection rate compared with the existing system. This work tries to reveal that compared to the existing application-oriented algorithms [30,31,39,40,41,42,43,44], RSC performs better in the event-based and time-critical applications. 

The scenarios and the number of rounds used for evaluating the performance of RSC was inherited from fan-shaped clustering and the only difference is both the algorithms were implemented and evaluated for different statistical measures [35,36,37,38,39,40,41].

Since RSC and FSC were evaluated with same set of scenarios, the improvement in percentage may be addressed stochastically. But the improvement varies according to the parameter selected for comparison. Here, the number of nodes alive are 16% more in RSC and from the energy conservation point of view, RSC produces a 34% improvement, whereas, RSC produces 45% improvement in statistical measures mean and median. All the above results are obtained when compared with FSC. Hence, the results were not addressed stochastically. In future, this might be addressed with various quadrants.

## 6. Conclusions and Future Scope

This paper proposes angular inclination-based RSC and routing. The network area is considered as a circular area, and it is divided into equal-sized virtual concentric rings based on the radius of the circle. Each ring is further divided into equal-sized clusters, which increases gradually from the inner layer to the outer layer. The CH is selected based on the nearest distance to the midpoint of the cluster. Furthermore, the CH is updated based on the number of live nodes and the residual energy to ensure load balancing. The number of nodes and packet collection rates are high because RSC is implemented for large-scale networks. To identify the optimal aggregation measures for large-scale applications, the statistical measures are considered with the objective of a high packet collection rate with reduced reclustering frequency. There is a tradeoff between the transmission distance and the range when the quantity of live nodes and routing is considered. It was found that the RSC outperformed the existing FSC in network energy conservation.

Even though the present work yielded better results compared to the existing state-of-the-art algorithms, static random deployment with the manual change of quadrants is to be implemented in future. For real-time applications, it would be more useful if the quadrant is changed dynamically. This work addresses only flat surface deployment. In future, it should be extended to address regions with sloppy surfaces to bring realistic results. In addition to this, security of sensor networks and secure data transmission has to be taken into consideration for further improvement.

## Figures and Tables

**Table 1:** Simulation parameters used in RSC.

Parameters Used	Values
Area of deployment (x,y)	500 m ∗ 500 m
Number of nodes	1000
Coordinate of the sink node ($S_{x}\;,\;S_{y)}$	(260, 260) m
The initial energy of each sensor	2 J (Joule)
$E_{elec}$	50 nJ/bit
$\varepsilon_{fs}$	10 pJ/bits/m^2^)
$\varepsilon_{mp}$	0.0013 pJ/bits/m^4^)
$E_{DA}$	5 nJ/(bit ∗ signal)
$d_{0}$	$\surd \frac{\epsilon_{fs}}{\varepsilon_{mp}}$
x,y coordinates of midpoint of grid	$S\left(p_{x},p_{y}\right)$
x,y coordinates of all nodes	A(x,y)
Number of rounds K	500
Control packet size $P_{ctrl}$	200 bits
Data packet size $P_{data}$	3000 bits
